# Hydrogen Peroxide Release Kinetics of Four Tooth Whitening Products—In Vitro Study

**DOI:** 10.3390/ma14247597

**Published:** 2021-12-10

**Authors:** Susana Dias, António Mata, João Silveira, Ruben Pereira, Angelo Putignano, Giovanna Orsini, Riccardo Monterubbianesi, Duarte Marques

**Affiliations:** 1 Oral Biology and Biochemistry Research Group, Faculdade de Medicina Dentária, Universidade de Lisboa, 1950-044 Lisboa, Portugal; dias.susana@campus.ul.pt (S.D.); antonio.mata@fmd.ulisboa.pt (A.M.); silveira@campus.ul.pt (J.S.); ruben-pereira@campus.ul.pt (R.P.); 2 LIBPhys-FCT UID/FIS/04559/2013, Faculdade de Medicina Dentária, Universidade de Lisboa, 1950-044 Lisboa, Portugal; 3 Department of Clinical Sciences and Stomatology, Polytechnic University of Marche, 60100 Ancona, Italy; a.putignano@staff.univpm.it (A.P.); g.orsini@staff.univpm.it (G.O.); r.monterubbianesi@pm.univpm.it (R.M.); 4 Postgraduate Program in Prosthodontics, Faculdade de Medicina Dentária, Universidade de Lisboa, 1950-044 Lisboa, Portugal

**Keywords:** tooth bleaching, hydrogen peroxide, release kinetics, tooth whitening, in vitro

## Abstract

Tooth whitening efficacy can be influenced by several factors, of which concentration and application time are two of the most important. This in vitro study aimed to evaluate the initial content and release kinetics of the hydrogen peroxide (HP) content, or the carbamide peroxide (CP) content as converted to its HP equivalent, of four tooth whitening products with different concentrations (6% HP, 16% CP, 10% CP, and 5% CP). Titrations with Cerium Sulphate IV were performed to determine HP concentration. HP release kinetics were evaluated by a spectrophotometric technique. The results were expressed as the mean values and 95% confidence interval of the percentage of hydrogen peroxide content during release kinetics. One sample *t*-test, one-way ANOVA, Tukey post hoc testing, and Pearson correlation testing were used, as appropriate, with a significance level of α = 0.05. The concentration of titrated HP was higher than that indicated by the manufacturers in all tested products (*p* < 0.01). At the minimum application times indicated by the manufacturers, all products released at least 85% of HP content; the gel containing 10% CP registered the lowest release at 85.49 (81.52–89.46). There was a significant HP release in all products during the application times indicated by the manufacturers. Further studies are needed to assess in vitro release kinetics.

## 1. Introduction

The number of dental whitening treatments performed worldwide has increased in recent years due to public demand, and the number of tooth whitening products available on the market has also increased [1,2]. These products are mostly based on carbamide peroxide (CP) or hydrogen peroxide (HP), and may differ in concentration, formulation, application times, and techniques [3,4,5,6].

Although several hypotheses explain the action of HP as a bleaching agent [7,8], the most accepted current hypothesis is that HP does not induce significant changes in the relative organic and inorganic content of tooth enamel; rather, it whitens teeth by oxidizing their organic matrices [7,9]. The free radicals released split the cyclic carbon rings of pigmentation molecules with high molecular weight into linear ones that are simpler to remove from tooth structure [10,11]. These molecules, in turn, condition a greater reflection of light, thus giving the perception of brighter teeth [11,12].

Two of the most important properties of a whitening product are the degree of diffusion through tooth structure and the ability to react with chromophore molecules. The selection of a bleaching product depends not only on efficacy, release rate, or the potential to cause damage to surrounding structures, but also on the differential diagnosis of tooth discoloration and the choice of technique [13,14,15,16].

Since Haywood and Heymann’s description, in 1989, of the original vital bleaching nightguard technique, which involved applying a 10% CP gel to an individual tray, manufacturers have launched products into the market with higher concentrations, claiming a faster treatment effect [17,18].

However, in 2011, a European directive considered tooth whitening products containing up to 6% HP or equivalent content as cosmetic products. This directive led to a change in the industry, with the commercialization of different formulations and application methods that complied with the legal limit and maintained a comparable efficiency [19,20].

Additionally, a new molecule, NOVON^®^, was added to some whitening products which, according to previous studies, allows a significant increase in pH and consequently increases the release of free radicals during the application time, without chemical or microscopical change in enamel surface. This new formulation differs from other hydrogen or carbamide peroxide-based products, in that it contains sodium tripolyphosphate [21,22].

Thus, the purpose of this in vitro study was to evaluate the initial content and release kinetics of HP in four tooth whitening products with different concentrations (6% HP, 16% CP, 10% CP, and 5% CP) containing NOVON^®^ in their formulation. The null hypotheses are: (1) there are no differences in titrated HP content compared to the manufacturers’ claimed concentrations in their whitening products, and (2) the whitening products release at least 85% of the HP content at the application times indicated by the manufacturers.

## 2. Materials and Methods

### 2.1. Initial Titration

**Figure 1:**
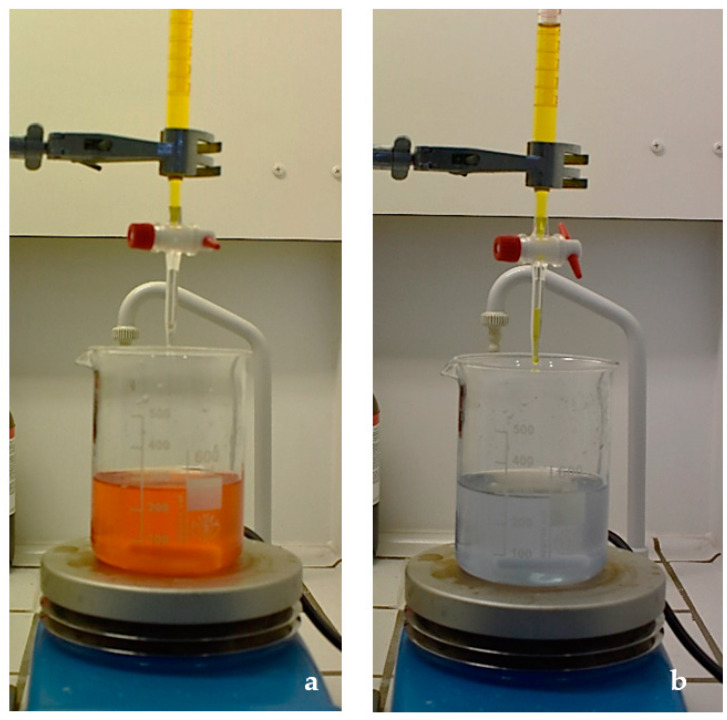
Titration with 0.1 M cerium (IV) sulfate before (**a**) and after (**b**) the equivalence point.

Three different batches of each product were analyzed and distributed in the following groups: group 1—6% HP; group 2—16% carbamide peroxide (CP); group 3—10% CP; and group 4—5% CP (White Dental Beauty^®^, West Yorkshire, UK) (Table 1). Titrations were performed with Cerium Sulphate IV (CS) to determine the HP concentration [23,24]. The samples were weighed in a 600 mL beaker on an analytical balance with ± 0.1 mg sensitivity (Metler Toledo AB54, Toledo, Spain). Each sample was diluted in 225 mL of deionized water; 25 mL of a solution of 5.0 M sulfuric acid (H_2_SO_4_) were added while stirring over a stir plate (200–300 rpm); and 10 drops of ferroin indicator solution were added (Fluka, Switzerland), giving the solution a dark orange color. The samples were titrated with a 0.1 M Cerium Sulfate IV solution (CeSO_4_·4H_2_O, # 31606, Sigma-Aldrich, Darmstadt, Germany). At the equivalence point, the sample changed from dark orange to pale blue (Figure 1). The relationship between the volume of CS used in the titration of the sample and the sample weight (SW) was expressed in a formula and used to determine the percentage of HP (% by weight) that was recovered in each sample (formula (1)):

% HP (% by weight) = CS volume (mL) × 0.17 SW (g)(1)

CP concentration was converted to its HP equivalent using the following formula (Formula (2)) [25]:

(2) $$\%\;\mathrm{CP}\;\left(w/w\right)=\frac{{\%\;H}_{2}O_{2}\;}{0.362}$$

The initial concentration of each batch of the whitening products was determined until a minimum of three replicates was obtained with results within an interval of 0.5% [26].

### 2.2. Release Kinetics of HP

**Figure 2:**
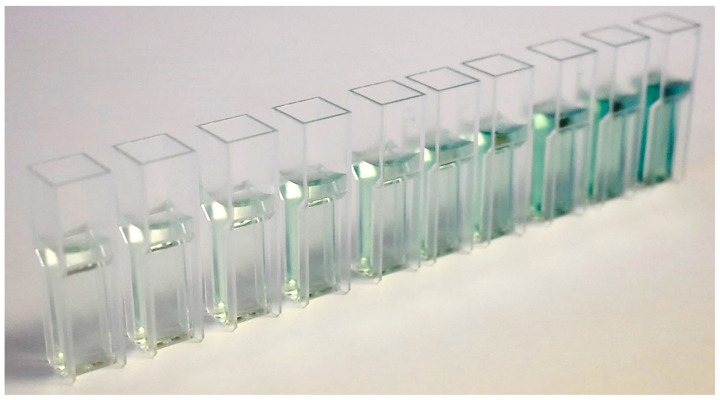
Example of the calibration curve solutions for the ABTS technique used. Calibration curves were from fresh solutions prepared daily.

The release kinetics of HP was evaluated by a spectrophotometric technique previously established, which uses 2,2’-Azino-bis (3-ethylbenzothiazoline-6-sulfonic acid) diammonium salt (ABTS) as a chromogen [26,27]. In each test, approximately 300 mg of gel were weighed and scattered on the bottom of a petri dish (VWR, Sarstedt, Germany), previously weighed. Immediately, 15 mL of distilled water were added to the peripheral area of the petri dish and placed on an orbital shaker (Kottermann 3047, Uetze, Germany) at a speed of 50 rpm. At predetermined times (0, 1, 2, 3, 4, 5, 10, 15, 20, 30, 40, 60, 90, 120, 150, 180, and 240 min) that accorded with the application times indicated by the manufacturers of each of the products, a 100 μL sample was collected and replaced in deionized water using a micropipette (P100 Eppendorf, Sigma-Aldrich, Darmstadt, Germany). The samples were analyzed in a spectrophotometer (M501 single beam, CAMSPEC, Leeds, United Kingdom) at 420 nm wavelength to determine HP content by a peroxidase-based colorimetric method. A phosphate-based buffer solution was made from two buffer solutions (87.9% by volume of 0.05 M KH_2_PO_4_ and 12.1% by volume of 0.05 M Na_2_HPO_4_), with a pH = 6.0. From this solution, a solution of ABTS 2.08 M and a solution of horseradish peroxidase 400 U /mL were elaborated. The final volume present in the microcuvette (MonoLab, Carpi, Italy) was 1.5 mL (1.44 mL ABTS buffer solution + 30 μL peroxidase buffer solution + 30 μL sample). For the calibration of the spectrophotometer (M501 single beam, CAMSPEC, Leeds, United Kingdom), the calibration curves were drawn up on the same day, with 10 calibration points, being accepted as valid with a confidence coefficient equal to or greater than 99% (Figure 2). The calibration points shown represent the following concentrations: 0, 50, 100, 200, 300, 400, 600, 800, 1000, and 1500 μM PH. Ten samples of each batch were analyzed (*n* = 30 per group) in the predetermined time periods.

### 2.3. Statistical Analysis

The titration results were expressed as the mean values and 95% confidence interval (CI) of the percentage of titrated HP. The percentage of HP released into the aqueous medium were expressed as the mean values and 95% CI, considering 100% as the initial titrated value. One sample *t*-test, one-way ANOVA, Tukey post hoc testing, and Pearson correlation tests were used, as appropriate, with a significance level of α = 0.05. 

Data analysis and results were calculated using statistical pack SPSS (IBM Statistics v.24, Inc., Chicago, IL, USA).

## 3. Results

### 3.1. Initial Titration

The concentrations of titrated HP were higher than those reported by the manufacturers for all tested products (*p* < 0.01) (Table 2). No significant differences were detected between the batches for each group.

### 3.2. Kinetic Release of HP

**Figure 3:**
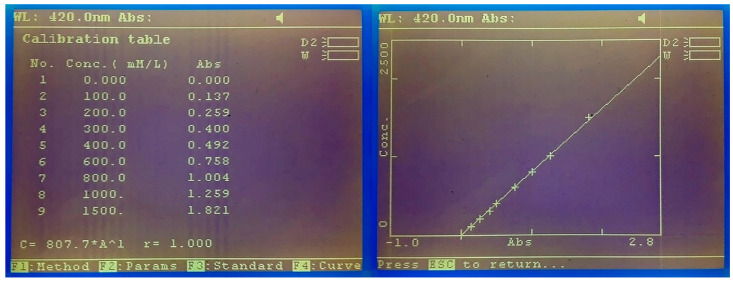
Example of the calibration curve values and graph representation for the ABTS technique used. Absorption readings were recorded at 420 nm and confidence coefficients >0.99 were accepted as valid.

**Figure 4:**
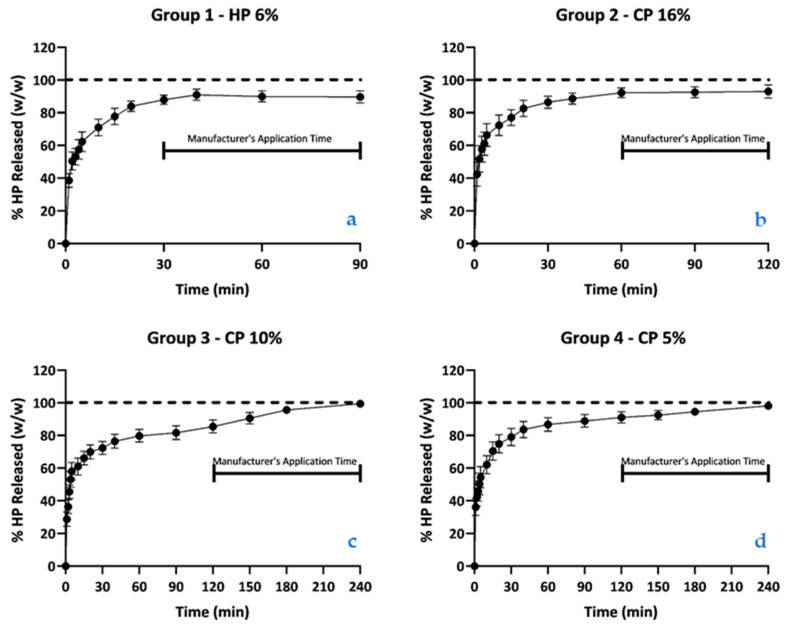
(**a**–**d**). Graph with the mean values and 95% CI of the percentage of HP released as a fraction of the titrated value, for the pre-determined times in (**a**) Group 1; (**b**) Group 2; (**c**) Group 3; and (**d**) Group 4 (*n* = 30 for each group).

Table 3 and Figure 3 and Figure 4 report the mean values and 95% CI of the percentage of hydrogen peroxide content during kinetic release into the aqueous medium of each bleaching product. The results show that the products exhibit an exponentially fast release of hydrogen peroxide into the aqueous medium during the initial 30 min, after which the release is attenuated to a plateau phase.

The percentages of HP released in the four groups, at the minimum and maximum application times, were compared. At the minimum application times, there were statistically significant differences in the percentages of HP released between Group 2 (16% CP) and Group 3 (10% CP). At the maximum application times, there were statistically significant differences between Group 1 (6% HP) and Group 3 (10% CP), between Group 1 (6% HP) and Group 4 (5% CP), and between Group 2 (16% CP) and Group 3 (10% CP).

## 4. Discussion

The first aims of this study were to assess the HP concentration in different whitening products and to determine any variability among different batches.

It was determined that HP content in the four groups was higher than reported by the manufacturers, thus rejecting the null hypothesis. The data are in accordance with earlier studies that evaluated different bleaching products, where the obtained values were also higher than those indicated by the manufacturers [14,26,28].

Previous studies reported that HP and CP concentrations decrease over shelf time, due to chemical degradation, with a greater effect in hydrogen peroxide-based products [10,29,30]. Bleaching products could present lower concentrations when compared to the manufacturers’ claimed concentrations due to HP and CP degradation over time [28,31,32]. Thus, a possible reason for the obtained data could be manufacturers’ overcompensation for degradation during shelf time. While some studies report that different concentrations and formulations can justify different clinical outcomes in tooth whitening treatments, caution should be exercised when assessing the present results, as the differences detected were small and may not be clinically significant or inconsistent with product efficacy [33,34].

According to the American Dental Association, HP release kinetics is an important parameter in determining the safety and effectiveness of an at-home bleaching agent, being related to the rate of degradation of an active agent in a specific period [32]. Our results suggest that at least 85% of total HP content is released when the manufacturers’ proposed minimum application times are attained, with faster kinetics in the initial 30 min followed by a sustained release until the maximum application times. These results could be explained by the new molecule sodium tripolyphosphate (NOVON^®)^ that is present in these products. NOVON^®^ can produce a significant increase in solution pH upon dilution, leading to an increase in oxygen-derived free radicals released into aqueous medium. Another study, comparing a 10% CP-based product with a 5% CP-based product with NOVON^®^, suggests that this new molecule can achieve similar whitening effects using significantly lower levels of hydrogen peroxide concentration. 

Although dental bleaching is an effective procedure, several authors have reported structural changes and possible damage to oral structure with excessive use, mainly side effects such as burns, transient sensitivity, reduction of microhardness, reduction of bond strength of restorations, and increase of roughness [35,36,37]. More recently, NOVON^®^-based products have been reported as not causing significant structural alterations to the surface of enamel when submitted to different concentrations of hydrogen peroxide; the use of antioxidants prior to bonding is able to reverse the compromised enamel-composite bond strength following bleaching with 5% and 10% CP [35,37].

When considering the obtained results, it was possible to observe that almost all HP content was released during the manufacturers’ recommended time intervals, although total HP release was never attained.

Our results suggest that extended application times may not be necessary for high release kinetics of these products. Clinical studies comparing the minimum and maximum wear time of each product should be carried out to assess the influence of application time on bleaching efficacy.

## 5. Conclusions

The HP content titrated was higher than indicated by the manufacturers for all products. All products released at least 85% of their HP content at the manufacturers’ minimum application times, suggesting that the application intervals proposed by the manufacturers are appropriate for increased efficiency. Further clinical studies are necessary to correlate release kinetics with tooth whitening efficacy.

## Figures and Tables

**Table 2:** Mean ± 95% CI concentration of HP detected in the bleaching products; *n* = 9.

Groups	Batches	% HP Reported by Manufacturer	Mean [95 CI] %HP Titrated
Group 1 (6% HP)	2219 3519 7619	6	6.32 [6.26–6.38]
Group 2 (16% CP)	1919 2719 7319	5.79	6.20 [6.17–6.23]
Group 3 (10% CP)	1619 2319 6719	3.62	3.77 [3.74–3.80]
Group 4 (5% CP)	1719 2819 5019	1.81	1.93 [1.90–1.96]

**Table 3:** Table with the mean values and 95% CI of the percentage of HP released as a fraction of the titrated value, at the corresponding minimum and maximum application time.

Groups	Mean [95 CI] %HP Released at Minimum Application Time	Minimum Application Time (min)	Mean [95 CI] %HP Released at Maximum Application Time	Maximum Application Time (min)
Group 1 (6% HP)	87.94 [85.14–90.74]	30	89.64 [85.94–93.32]	90
Group 2 (16% CP)	92.21 [89.28–95.14]	60	93.02 [88.96–97.08]	120
Group 3 (10% CP)	85.49 [81.52–89.46]	120	99.40 [98.02–100.78]	240
Group 4 (5% CP)	91.04 [87.56–94.52]	120	98.19 [96.78–99.60]	240

**Table 1:** Whitening products’ characteristics.

Whitening Product	Groups	Active Agent	Active Agent (%)	Application Time (min)
White Dental Beauty 6% HP	1	HP	6	30–90
White Dental Beauty 16% CP	2	CP	16	60–120
White Dental Beauty 10% CP	3	CP	10	120–240
White Dental Beauty 5% CP	4	CP	5	120–240

## Data Availability

Original data are available on request from the corresponding author.
